# Concurrent production of glycyrrhetic acid 3-*O*-mono-β-d-glucuronide and lignocellulolytic enzymes by solid-state fermentation of a plant endophytic *Chaetomium globosum*

**DOI:** 10.1186/s40643-021-00441-y

**Published:** 2021-09-15

**Authors:** Boliang Gao, Yiwen Xiao, Qian Zhang, Junru Sun, Zhibing Zhang, Du Zhu

**Affiliations:** 1 Key Lab of Bioprocess Engineering of Jiangxi Province, College of Life Sciences, Jiangxi Science and Technology Normal University, Nanchang, 330013 China; 2 Key Laboratory of Protection and Utilization of Subtropic Plant Resources of Jiangxi Province, Jiangxi Normal University, Nanchang, 330022 China

**Keywords:** Glycyrrhetic acid 3-*O*-mono-*β*-d-glucuronide, *Chaetomium globosum* DX-THS3, Solid-state fermentation, Lignocelluloses-degrading enzymes

## Abstract

Glycyrrhetic acid 3-*O*-mono-*β*-d-glucuronide (GAMG) as an important derivative of glycyrrhizin (GL) shows stronger biological activities and higher sweetness than GL. The biotransformation process is considered as an efficient strategy for GAMG production, due to its mild reaction, high production efficiency and environmentally friendly status. In this study, licorice straw was used for the first time as a medium for GAMG and lignocellulosic enzyme production via solid-state fermentation (SSF) of endophytic fungus *Chaetomium globosum* DX-THS3. The fermentation conditions including particle size, temperature, seed age, inoculum size, and moisture of substrate were optimized. Furthermore, additional nitrogen sources and carbon sources were screened for GAMG production by *C. globosum* DX-THS3 of SSF. Under optimal fermentation conditions, the percent conversion of glycyrrhizin reached 90% in 15 days, whereas the control needed 35 days to achieve the same result. The productivity of optimization (*P* = 2.1 mg/g/day) was 2.33-fold that of non-optimization (*P* = 0.9 mg/g/day). Meanwhile, high activities of filter paper enzyme (FPase) (245.80 U/g), carboxymethyl cellulase (CMCase) (33.67 U/g), xylanase (83.44 U/g), and *β*-glucuronidase activity (271.42 U/g) were obtained faster than those in the control during SSF. Our study provides a novel and efficient strategy for GAMG production and indicates *C. globosum* DX-THS3 as a potential producer of lignocellulolytic enzymes.

**Supplementary Information:**

The online version contains supplementary material available at 10.1186/s40643-021-00441-y.

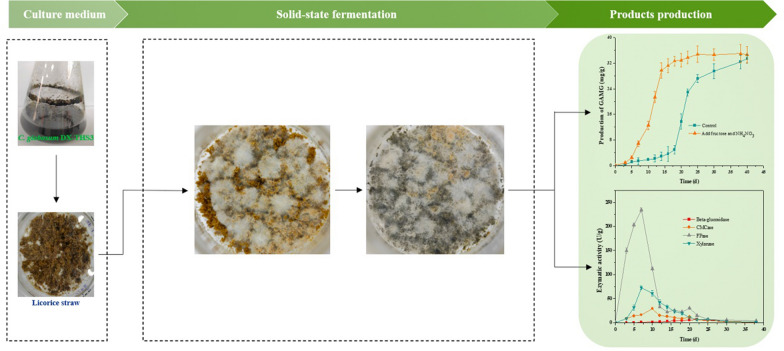

## Introduction

Glycyrrhizin (GL) is a major bioactive component of industrial crop *Glycyrrhiza* (Pandey and Ayangla 2017) with numerous valuable physiological properties, including antioxidative (Michaelis et al. 2011), antiviral (Ito et al. 1988), anticancer (Huang et al. 2016), and anti-inflammatory (Wang et al. 2015a) action. GL can efficiently inhibit the coronavirus disease (COVID-19) virus, and it is commonly considered a potential chemical for the cure of COVID-19 (Bailly and Gérard 2020; Murck 2020). However, GL has low absorption and strong adverse effects in humans and animals (Akao 2000; Wang et al. 2013). GL can be transformed into glycyrrhetic acid 3-*O*-mono-*β*-d-glucuronide (GAMG) (Additional file 1: Fig. S1), which possesses improved biological activities and safety and higher solubility, by hydrolysis of one of the terminal glucuronic acids of GL (Lin et al. 2009; Li et al. 2017). Furthermore, GAMG is about 5 and 1000 times sweeter than GL and sucrose (Mizutani et al. 1994), respectively, and tastes better (less aftertaste) than GL. Therefore, GAMG has valuable application, especially in the food industry. However, efficient approaches for large-scale GAMG production are unavailable thus far.

GAMG can be produced by chemical synthesis and biotransformation. However, chemical approaches generally present several disadvantages, including the requirement of strong and harsh conditions, ineffective costs, poor selectivity, and environmental pollution (Brieskorn and Lang 1978). Compared with chemical approaches, the biosynthesis of GAMG significantly has more potential and advantages because of its excellent substrate selectivity, high yields, mild reaction conditions, and eco-friendly status. The efficient biosynthesis of GAMG involves the use of *β*-glucuronidase (GUS) (Zou et al. 2013; Xu et al. 2018a, b; Wang et al. 2013; Park et al. 2005). At present, GUSs are mainly screened from microorganisms (Zou et al. 2013; Xu et al. 2018a, b; Wang et al. 2013; Park et al. 2005). However, most of reported GUSs exhibit a low hydrolytic selectivity and cause further transformation of GAMG to GA. On the other hand, GUS is generally prepared by microorganic fermentation. The isolation of GUS for biosynthesis of GAMG has several challenges, such as complicated processes and harsh cultivation conditions. Therefore, an efficient, simple, and feasible approach for the production of GAMG must be developed.

In general, most enzymes are produced by microorganic submerged fermentation (SF). GUS can also be produced by SF of *Aspergillus terreus* Li-20 (Xu et al. 2018a, b), *Streptococcus* LJ-22 (Park et al. 2005), and *Penicillium purpurogenum* Li-3 (Zou et al. 2013). However, their applications in the manufacturing industry have been met with several processing challenges, including low productivity, especially in terms of ineffective costs of the mass production of enzymes. Compared with SF, solid-state fermentation (SSF) can offer several advantages, including low equipment requirements, simple culture processing, and the use of widely available and inexpensive lignocellulosic residues (usually crop straws and wastes) as substrates (Pandey 2003). Therefore, SSF is very suitable to produce highly bioactive products, such as enzymes. Thus, the utilization of SSF for large-scale production of GAMG should be considered. However, to date, the use of SSF to produce GAMG has not been investigated.

Endophytic fungi are an ecological group of fungi present in living plant tissues without initiating any external symptoms. Endophytic fungi have attracted research attention because they can secrete a number of secondary metabolites, including bioactive chemicals (Suryanarayanan et al. 2012). In addition, endophytic fungi can utilize plant material, for example, crop straw, for use as a medium for SSF to produce valuable products, such as bioactive chemicals and enzymes (Prajapati et al. 2018; Natália et al. 2018; Deswal et al. 2011). In our previous study, an endophytic fungus *Chaetomium globosum* DX-THS3 was isolated from Dongxiang wild rice (*Oryza rufipogon* Griff.) (Wang et al. 2015b). Furthermore, a GUS with specificity and highly transformable GL to generate GAMG was screened from *C. globosum* DX-THS3 (Zhang et al. 2020), and genome analysis showed abundant genes coding lignocellulose-degrading enzymes harbored in the *C. globosum* DX-THS3 genome (unpublished). Therefore, in this study, *C. globosum* DX-THS3 was utilized with licorice straw as a medium for SSF. We aimed to i) investigate the feasibility of GAMG production by *C. globosum* DX-THS3 via SSF and provide an efficient strategy for large-scale production of GAMG; ii) analyze the lignocellulose-degrading enzymes, such as carboxymethyl cellulase (CMCase), FPase, xylanase, and GUS, during SSF to simultaneously obtain high-activity enzymes and demonstrate *C. globosum* DX-THS3 as a potential candidate for producing lignocellulose-degrading enzymes.

## Material and methods

### Chemicals and strains

Licorice straw was purchased from Inner Mongolia, China. Standard GL and GA samples (purity ≥ 98%) were purchased from Sigma Chemical Co. (USA). Standard GAMG (purity > 98%) was prepared and identified via carbon-13 nuclear magnetic resonance in our laboratory. Other chemicals and solvents (analytical grade) were purchased from Xilong Scientific Co., Ltd. (China). The endophytic fungal strain *C. globosum* DX-THS3 (CCTCC M2016005) was isolated from healthy Dongxiang wild rice in a nature reserve in Dongxiang county, Jiangxi province, China in our previous work and collected by Jiangxi Normal University, Nanchang, China.

### Production of GAMG by SSF

Licorice straw was dried for 24 h in a drying oven (50 °C) and then crushed to obtain < 3 mm grain diameter. *C. globosum* DX-THS3 was cultured in a 500-mL flask with 200 mL potato dextrose broth (PDB) at 28 °C on a rotary shaker at 150 rpm for seed broth preparation. Then, 2 mL seed broth was cultured in 15 g sterile licorice straw grain, which was pre-added with 5 mL sterile water at 28 °C for the production of GAMG. After 20 days of culture, 2 g of culture medium was ground and added to 100 mL water for the determination of GAMG using thin layer chromatography (TLC) and ultraperformance liquid chromatography (UPLC, Waters, USA).

### Optimization of SSF conditions

Strain DX-THS3 was cultivated in different fermentation conditions to detect the GUS activity and yield of GAMG for optimization of fermentation conditions after culturing for 20 days. First, 2 mL of seed broth was cultivated in licorice straw of different particle sizes (grain diameter: 0–0.25, 0.26–0.85, and 0.86–3.0 mm) at 28 °C, 15% of inoculum, straw: water = 1:3 and 72 h of seed age, respectively; For optimization of culture temperature, 24 °C, 28 °C, and 33 °C were used to culture strain DX-THS3, respectively, under 0.26–0.85 mm particle sizes of licorice straw, 15% inoculum and 72 h of seed age; To optimize seed age, different seed broths cultivated in PDB medium for 48, 60, 72, 84, 96, 108, 120, and 132 h were grown on licorice straw at 28 °C, 15% inoculum, 0.26–0.85 mm particle sizes and straw: water = 1:3; Then, different volumes of seed broths (15%, 20%, 25%, 30%, 35%, and 40%, v/w) in licorice straw were optimized for GUS and GAMA production by strain DX-THS3 of fermentation at 28 °C, 0.26–0.85 mm particle sizes, straw: water = 1:3 and 72 h of seed age; for exploration of the optimal water content of licorice straw, strain DX-THS3 were cultured on licorice straw containing different water contents (straw: water = 1:2, 1:2.5, 1:3, 1:3.5, m/m) at 28 °C, 0.26–0.85 mm particle sizes, 15% inoculum and 72 h of seed age; finally, 5 mg/g of additional nitrogen (NH_4_NO_3_, peptone, yeast powder, and yeast extract) and carbon sources (glucose, fructose, sucrose, lactose), were added to the licorice straw, respectively, for SSF of strain DX-THS3 under 28 °C, 0.26–0.85 mm particle sizes, 15% inoculum, 72 h of seed age and straw: water = 1:3. All cultivations were performed in triplicate.

### Determination of lignocellulosic enzyme activities

After 20 days of SSF or at different time points (shown in "Determination of total and reducing sugars" section), solid-state medium was sampled and dried by vacuum freeze drier (EYELA, Japan), 2 g of dried solid-state medium was ground by liquid nitrogen, and then dissolved in 15 mL sodium acetate (pH 5.0). The suspension was centrifuged at 4 °C and 10,000×*g*, and the supernatant was collected for the preparation of crude enzymes. Carboxymethyl cellulase (CMCase) was assayed in a reaction containing 1 mL of 2% carboxymethyl cellulose in sodium acetate buffer (pH5.0, v/v) and 1 mL crude enzymatic solution. After incubation for 30 min at 50 °C, the reaction was stopped by immersion in boiling water for 5 min and reducing sugar was detected by 3,5-dinitrosalicylic acid (DNS) method (Zhao et al. 2008) with glucose as the standard. FPase activity was analyzed using Whatman No. 1 filter paper (1 × 6 cm^2^, 50 mg) in a 2 mL total volume reaction containing 1 mL crude enzymatic solution and 1 mL sodium acetate (pH 5.0) for 60 min at 50 °C. Then, the reducing sugar was detected by DNS method. One unit (U) of CMCase and FPase activity was defined as the amount of enzyme that released 1 µmol glucose equivalent per minute. Xylanase activity was assayed in a solution containing 1% (w/v) xylan (1 mL) in 50 mM sodium acetate buffer (pH 5.0) and appropriately diluted crude enzyme (1 mL). After 10 min incubation at 50 °C, reducing sugar was detected by DNS method. One unit (U) of xylanase activity was defined as the amount of enzyme that released 1 µmol xylose equivalents per minute. GUS activity was detected based on the release of ρ-nitrophenol (ρNP) from the ρ-nitrophenyl-β-d-glucopyranoside (ρNPG) substrate, and the absorbance was read at 430 nm. The assayed reaction containing 1 mL crude enzyme and 1 mL ρNPG (5 mM) was incubated for 10 min at 50 °C and then stopped by 3 mL 0.5 M sodium carbonate. One unit (U) of GUS activity was defined as the amount of enzyme that released 1 µmol ρNP equivalent per minute.

### Determination of total and reducing sugars

Two grams of different dry solid-state mediums from 0, 3, 5, 7, 10, 12, 14, 16, 18, 20, 22, 25, 30 and 38 days of SSF were sampled, ground by liquid nitrogen, and then dissolved in 15 mL water, respectively. The suspension was centrifuged at 4 °C and 10,000×*g*, and the supernatant was collected for sample preparation. The total sugar of solid-state medium was determined in accordance with the phenol sulfuric acid method (Masuko et al. 2005), whereas reducing sugars were quantified by the DNS method.

### Determination of GUS activity

Two grams of dry solid-state medium was sampled at 0, 3, 5, 7, 10, 12, 14, 16, 18, 20, 22, 25, 30 and 38 d of SSF, ground by liquid nitrogen, and then dissolved in 15 mL sodium acetate (pH 6.0), respectively. The suspension was centrifuged at 4 °C and 10,000×*g*, and the supernatant was collected for the preparation of crude enzymes. GUS activity was assayed by the reaction containing 200 µL crude enzymatic solution and 800 µL GL solution (2 g/L). Then, the reaction was incubated at 45 °C for 1 h and disrupted by boiling water and analyzed by UPLC. One unit (U) of GUS activity was defined as the amount of enzyme that released 0.1 µmol GAMG equivalent per minute.

### Determination of product and yield

For determination of the concentration and yield of product, solid-state medium was sampled at different time point (0, 3, 5, 7, 10, 12, 14, 16, 18, 20, 22, 25, 30 and 38 d), then was dried by vacuum freeze drier, 2 g of dry medium was ground by liquid nitrogen, and dissolved in 15 mL water, respectively. The resultant suspension was centrifuged at 4 °C and 10,000×*g*, and the supernatant was collected and diluted to 100 mL using water for sample preparation. Then, GAMG and GA were determined by an ACQUITY UPLC (Waters, USA) instrument using a C18 column (4.6 mm × 250 mm, 5 µm, InertSustain, Japan) and UV detector, at the detection wavelength of 254 nm, injection volume of 10 µL, flow rate of 1.0 mL/min, and mobile phase MeOH–0.5% acetate (80:20).

The concentration of GAMG in samples (defined as *C*_s_ (mg/mL)) was determined by UPLC with a standard curve. The concentration of GAMG per gram of solid-state medium (defined as *C*_ssf_ (mg/g)) was detected by *C*_ssf_ = (*C*_s_ × 100)/2. The production of GAMG by SSF (defined as *Y* (mg/g)) was quantified by *Y* = C_ssf_ × *m*/*m*_0_, whereas *m*_0_ is the weight (g) of initial substrate (licorice straw, 15 g in this study), and *m* is the weight (g) of solid-state medium after fermentation. The productivity of GAMG by SSF (defined as *P* (mg/g/day)) was shown by *P* = Y/day.

## Results and discussion

### Production of GAMG by *C. globosum* DX-THS3 using licorice straw as substrate

**Fig. 1:**
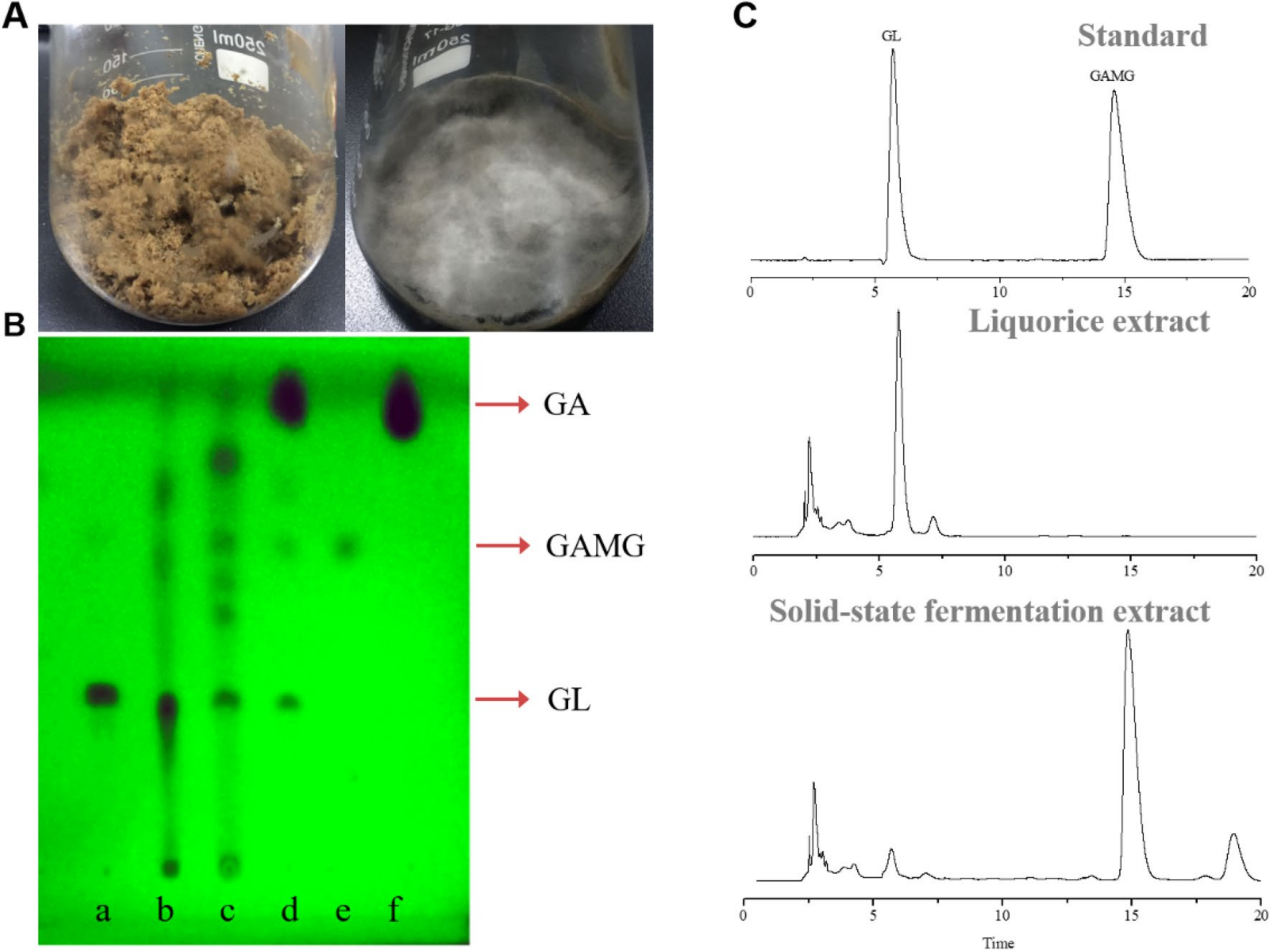
*C. globosum* DX-THS3 can grow on licorice straw for GAMG production. Licorice straw was used as a medium for *C. globosum* DX-THS3 growth (left, **A**). Depth fermentation was performed after 20 days (right, **B**), and the product was analyzed by TLC (B, lane a: standard of GL; lane b: extract of licorice straw; lane c: extract of solid-state medium; lane d: mix standard of GL, GAMG and GA; lane e: standard of GAMG; lane f: standard of GA) and UPLC analyses (**C**)

GAMG is an innovative functional sweetener with higher sweetness and stronger pharmacological activity than GL (Lin et al. 2009; Li et al. 2017; Mizutani et al. 1994). Biocatalysis of GAMG is a more environment-friendly and efficient than the chemical method (Brieskorn and Lang 1978). To date, two main strategies of bioconversion were investigated for GAMG production. One is the two-stage strategy for GAMG production, that is, GUS was firstly produced by fermentation of filamentous fungus such as *P. purpurogenum* Li-3 (Zou et al. 2013) and *Talaromyces pinophilus* (Xu et al. 2018a, b), and then GAMG was produced by bio-transforming GL with GUS. However, the production of GAMG was very low and unstable because of low biomass and GUS yield in the fermentation process by wild filamentous fungi. To address these issues, expression of recombinant GUS in *Escherichia coli* BL21 or *Pichia pastoris* were investigated by previous studies (Xiang et al. 2010; Qi et al. 2011). Another strategy used whole-cell biocatalysts for transformation of GL to produce GAMG. Whole-cell biocatalysts, including wild-type *P. purpurogenum* Li-3 and recombinant strains *E. coli BL21* and *P. pastoris* were used for GAMG production in an ionic liquid/buffer biphasic system (Chen et al. 2012). And the yield of GAMG reached at 2.62 g/L after 62 h by using this approach. Although there are important discoveries revealed by these studies, there are also limitations for the industrial-scale application of GAMG. First, production of GUS was very low due to unstable strain and low efficiency of GUS. Second, high instrumentation costs were needed during these processes. Third, considerable operator skill requirements must for performance. Overall, a novel strategy for high-efficient production of GAMG should be further developed. In our previous studies, an endophytic fungus *C. globosum* DX-THS3 was isolated from Dongxiang wild rice (Wang et al. 2015b), and a GUS (cg-GUS) with specificity and highly transformable GL to generate GAMG was screened from this strain (Zhang et al. 2020). In this work, strain DX-THS3 was cultivated in licorice straw to produce GAMG by SSF (Fig. 1A). After 20 days of cultivation, *C. globosum* DX-THS3 covered almost all of the licorice straw (Fig. 1A, right). Solid-state medium was sampled, and the product was analyzed by TLC to detect the GAMG. As shown in Fig. 1B, only GL was detected in the licorice straw (b), but after 20 days of fermentation, considerable GAMG was detected using TLC (c). UPLC analysis was also performed for identification and further confirmation of these products. Our results confirmed that GAMG was the product in SSF (Fig. 1C). Thus, our results show that GL of licorice straw can be bio-transformed to produce GAMG using licorice straw as a substrate by *C. globosum* DX-THS3. Comparing to previous works, this strategy has several advantages of low equipment requirements, simple culture processing, and low costs. To our knowledge, this work is the first to report the GAMG production by SSF using endophytic fungi. Meanwhile, this research also contributes to the application of endophytic fungi as potential industrial strains in food, biopharmaceutical, and biotechnological industries.

### Optimization of SSF conditions for GAMG production by *C. globosum* DX-THS3 using licorice straw as substrate

**Fig. 2:**
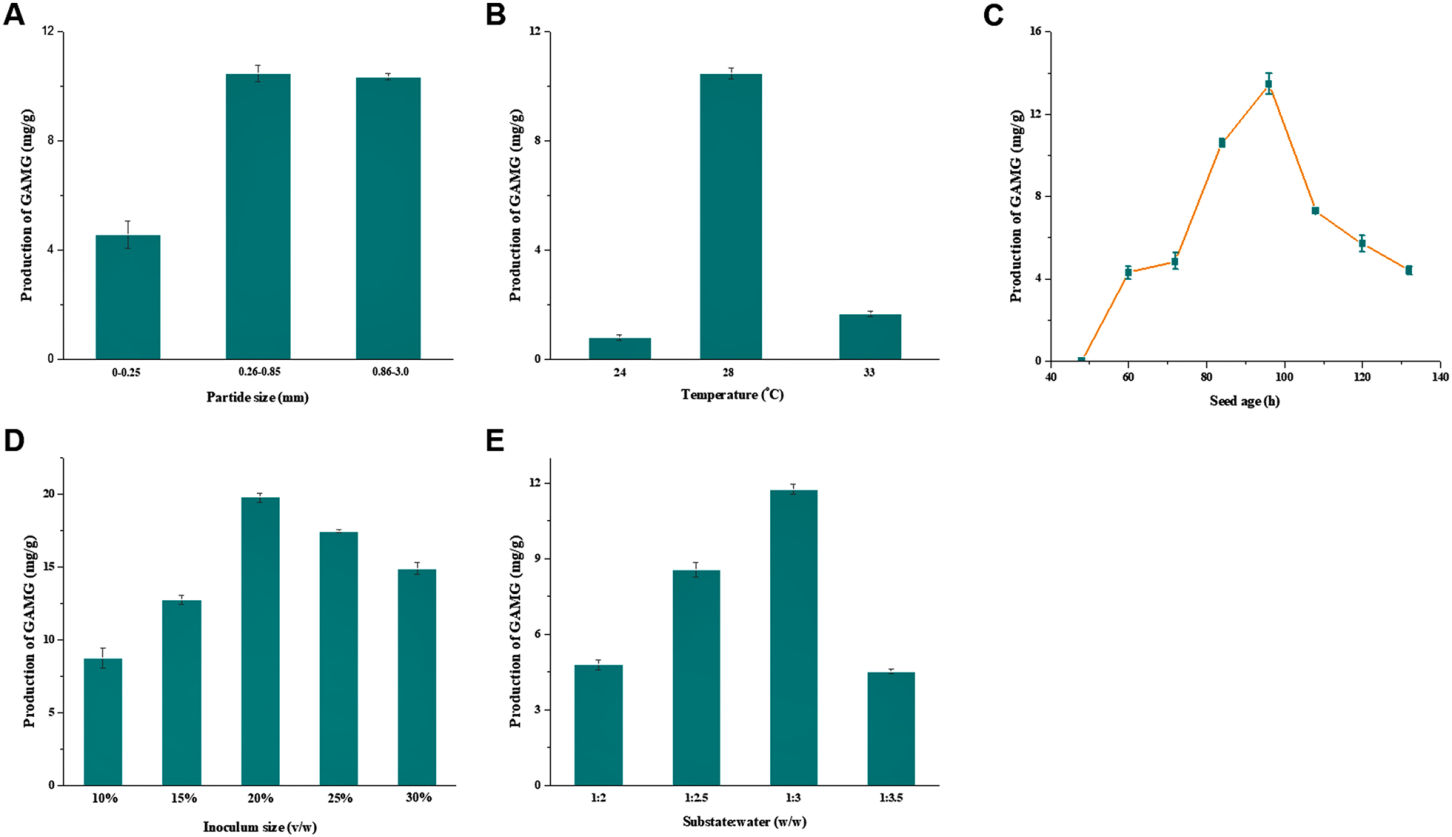
Optimization of fermentation conditions, including 0–0.25, 0.26–0.85, and 0.86–3.0 mm of particle size assessed at 28 °C, 96 h of seed age, 15% inoculum and substrate:water = 1:3 (**A**), 24 °C, 28 °C, and 33 °C of temperature assessed at 96 h of seed age, 15% inoculum, substrate:water = 1:3 and 0.26–0.85 mm of particle size (**B**), 48, 60, 72, 84, 96, 108, 120, and 132 h of seed age assessed at 28 °C, 15% inoculum, substrate:water = 1:3 and 0.26–0.85 mm of particle size (**C**), 15%, 20%, 25%, 30%, 35%, and 40% of inoculum size assessed at 28 °C, 96 h of seed age, substrate:water = 1:3 and 0.26–0.85 mm of particle size (**D**, v/w), and 1:2, 1:2.5, 1:3, 1:3.5 of substrate:water assessed at 28 °C, 15% inoculum, 96 h of seed age and 0.26–0.85 mm of particle size (**E**)

The fermentation conditions can markedly influence the products (not only the kinds, but also the yields) when fermenting using microorganisms (Singh et al. 2017). Thus, we optimized the SSF conditions for GAMG production by *C. globosum* DX-THS3 using licorice straw as substrate in the present study. Some special fermentation conditions of SSF, such as the particle size of substrate, initial moisture content of medium should be primarily considered. The particle size of substrate was rarely considered in optimization fermentation conditions of SSF. However, from a few previous studies, particle size of substrate also has important effect on products production by SSF, especially for lignocellulolytic enzymes production (Almeida et al. 2019; Botella et al. 2007; Yang et al. 2006). Yang et al. (2006) produced xylanase by *Paecilomyces thermophila* J18 on wheat straw in solid-state fermentation, and their results shown that highest xylanase was produced by the wheat straw of particle size 0.3–0.45 mm, whereas lower activities were produced on the wheat straw of other sizes. Thus, we first investigated the effect of particle size of licorice straw on GAMG production by *C. globosum* DX-THS3. As shown in Fig. 2A, our results indicated that too small medium particle size (4.56 mg/g, yield of GAMG, Y; particle size < 0.25 mm) of licorice straw was not better than the medium particle sizes (*Y* = 10.47 mg/g; 0.26–0.85 mm of particle size) and large particle size (*Y* = 10.35 mg/g; 0.86–3.00 mm of particle size) for the production of GAMG by *C. globosum* DX-THS3. This result ties well with previous studies wherein oversized or extremely small particle size of substrate is unsuitable for SSF using fungi (Almeida et al. 2019; Botella et al. 2007; Yang et al. 2006). Oversized particles can affect substrate release, especially that of lignocellulose, and significantly reduce the contact area of fungi with the substrate (Pandey 2003). On the other hand, an extremely small particle size can influence fungal growth (Pandey 2003). Water and substrate powder will mix to form a tight bulk or pellet, which not only significantly reduces oxygen transfer, but also obstructs in-depth growth of fungal mycelium. Comparing to particle size, the important effect of initial moisture content on products production by SSF was widely demonstrated in many previous studies (Almeida et al. 2019; Botella et al. 2007; Yang et al. 2006; Ajijolakewu et al. 2016; Nutongkaew et al. 2019; Ezeilo et al. 2019). Our results show that 1:3 solid–liquid ratio (75% of moisture, *Y* = 11.75 mg/g, Fig. 2E) was the optimal condition for the production of GAMG by SSF using *C. globosum* DX-THS3. These findings are consistent with previous studies (Almeida et al. 2019; Botella et al. 2007; Yang et al. 2006; Ajijolakewu et al. 2016; Nutongkaew et al. 2019; Ezeilo et al. 2019) showing that medium water content (about 70%) is required in SSF.

Furthermore, other general fermentation conditions, including temperature, seed age, and inoculum size were optimized. Our results show that 28 °C (*Y* = 10.47 mg/g, Fig. 2B), 96 h seed age (*Y* = 13.46 mg/g, Fig. 2C), and 20% inoculum size (*Y* = 19.78 mg/g, v/w, Fig. 2D) were the optimal conditions for the production of GAMG by SSF using *C. globosum* DX-THS3. The incubation temperature is one of the most vital parameters influencing efficacy of SSF. Deswal et al. (2011) showed that the highest CMCase (71.70 U/g), FPase (3.49 U/g) and β-glucosidase (53.68 U/g) activities were recorded for *Formitopsis* sp. RCK2010 cultivated on wheat bran at 30 °C. *Trichoderma asperellum* USM SD4 which grew on oil palm empty fruit bunch showed a lower optimum temperature of 27 °C for xylanase production (Ajijolakewu et al. 2016). In fact, these basic findings revealed optimum growth temperatures between 25 and 30 °C for products production by SSF of filamentous fungi (Almeida et al. 2019; Botella et al. 2007; Yang et al. 2006; Ajijolakewu et al. 2016; Nutongkaew et al. 2019; Ezeilo et al. 2019). Furthermore, from our work and previous studies, it must be pointed out that filamentous fungi are better than other microorganism, such as bacteria, for production of products by SSF due to their optimal incubation temperature closing to room temperature. In addition, most literature data concerning the influence of inoculum size on fungal products production by SSF cite the use of spores (Zhang and Sang. 2012; Xu et al. 2018a, b). Thus, it is difficult to compare these data with results of our study. Fortunately, *C. globosum* DX-THS3 can grow in PDB to generate uniform pellet, then was inoculated into licorice straw with same number of fungal pellets per 1 mL as far as possible. Similar to the present study, there are some studies investigated the effect of inoculum size on products production by using mycelial pellets, such as Zilly et al. (2012) used different mycelial pellets of 10 mm diameter as inoculum to obtain hydrolytic enzymes from *Pleurotus pulmonarius* by SSF using wheat bran as substrate. From the above discussion, the conclusion can be reached that fermentation conditions have significant effect on fungal products production by SSF.

### High activity of lignocellulosic enzymes during the degradation of licorice straw by *C. globosum* DX-THS3

**Fig. 3:**
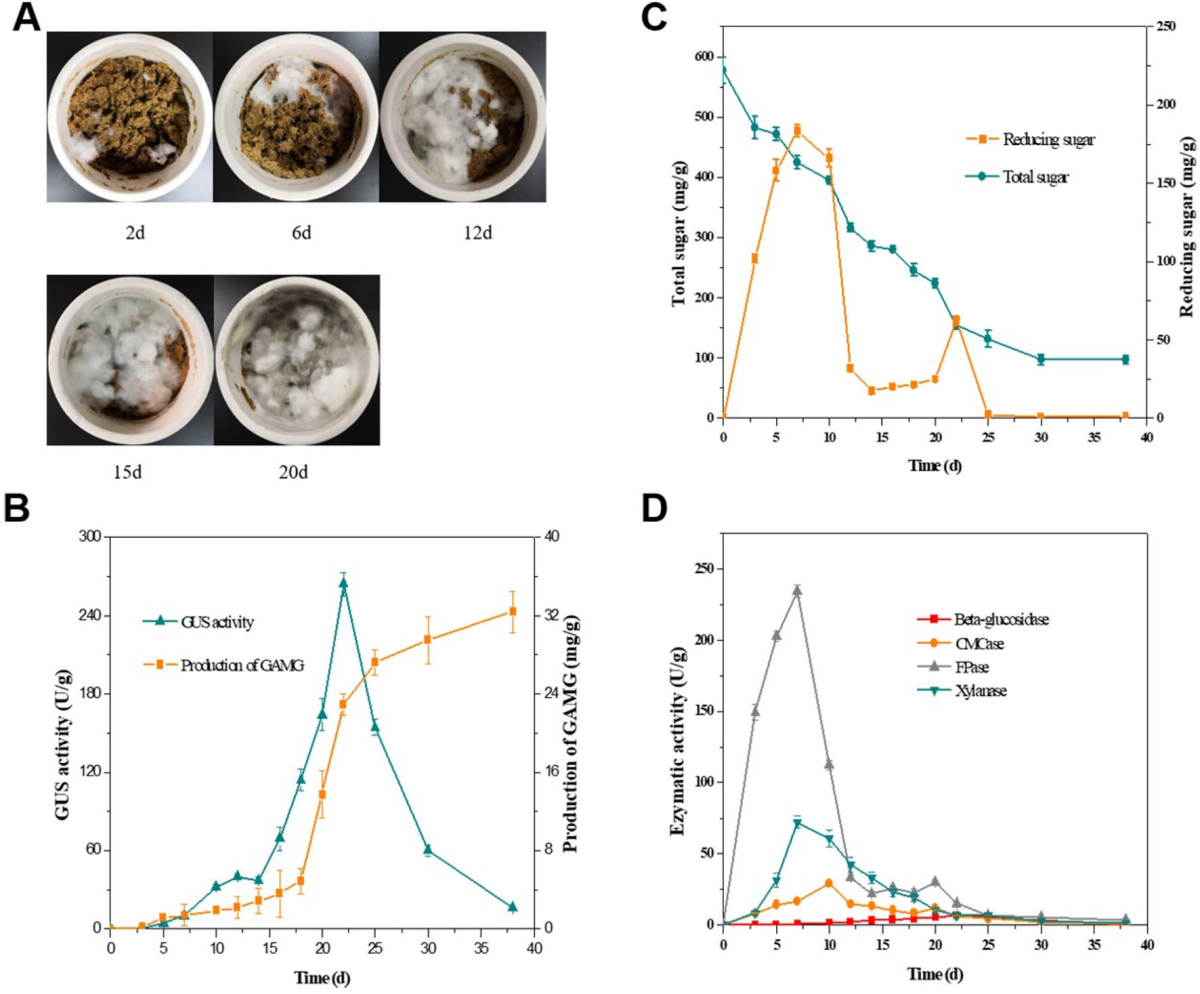
GUS activity, GAMG, reducing sugar, total sugar, and lignocellulose-degrading enzymatic activity profiles of *C. globosum* DX-THS3 under optimal SSF conditions. **A** *C. globosum* DX-THS3 was grown on licorice straw for depth fermentation. **B** GUS activity and yield of GAMG profile; **C** reducing sugar and total sugar profile; **D** CMCase, FPase, β-glucosidase, and xylanase profile

*Chaetomium globosum* DX-THS3 was first cultured in pre-treated licorice straw under 0.26–0.85 mm licorice straw, 28 °C, 96 h seed age, 20% inoculum size, and 1:3 solid–liquid ratio of fermentation conditions to further investigate the feasibility of this novel strategy for GAMG production. *C. globosum* DX-THS3 mycelium was grown slowly in the early stage of fermentation (0–6 days). Then, *C. globosum* DX-THS3 was gradually grown fast in the middle stage of fermentation (7–12 days), and the mycelium covered the licorice straw after 20 days of fermentation (Fig. 3A). The yields of GAMG by *C. globosum* DX-THS3 using licorice straw as substrate were analyzed during SSF to detect the production of GAMG by *C. globosum* DX-THS3. The production of GAMG was extremely low in the early and middle stages (0–18 days) of SFF (Fig. 3B). Until 18 days of fermentation, the yield of GAMG significantly increased. Our results demonstrate that the yield of GAMG reached 13.73 mg/g after 20 days, and the percent conversion of GL reached 90% (Y = 31.5 mg/g) after about 33 days of SSF (Fig. 3B). Meanwhile, GUS activity was also determined during SSF of strain DX-THS3. In the earlier stage of SSF (0–18 days), GUS activity was very low. Until 20 days of SSF, GUS activity was fast increased and highest activity was detected at 22 days of SSF (Fig. 3B). Furthermore, the total and reducing sugars were detected during SSF to investigate the utilization of carbon source by *C. globosum* DX-THS3. The total sugar of substrate continuously decreased during 0–30 days of SSF and stabilized afterward (Fig. 3C). Thus, the growth period of *C. globosum* DX-THS3 was mainly at 0–30 days of SSF. The reducing sugar increased rapidly at the early stage of SSF (0–7 days), and the highest concentration was detected at 7 days. Then, the reducing sugar was largely utilized by *C. globosum* DX-THS3 (Fig. 3C). The reducing sugar slowly increased again at the middle stage of SSF (20–22 days, Fig. 3C). The corresponding enzymatic activities, including those of CMCase, FPase, β-glucosidase, and xylanase, were analyzed during SSF to investigate the variation in reducing sugar during SSF by *C. globosum* DX-THS3. Lignocellulosic enzymatic activities were observed at the early stage of SSF, and the highest activities of CMCase (29.17 U/g), FPase (234.63 U/g), and xylanase (72.52 U/g) were detected at 10, 7, and 7 days of SSF, respectively (Table 1 and Fig. 3D). Meanwhile, our results showed low β-glucosidase activity during SSF of *C. globosum* DX-THS3, whereas 6.81 U/g enzymatic activity was detected at 22 days of SSF. Close correlation between these enzymes with the trend of total and reducing sugar can be concluded from our results (Fig. 3C and D) and previous studies (Nutongkaew et al. 2019; Ezeilo et al. 2019). The trend of reducing sugar is positive correlation with variation of lignocellulolytic enzyme activities during SSF by *C. globosum* DX-THS3, and it’s also ties well with older works. When comparing our results to those of older studies, it must be pointed out that unexpected increment of reducing sugar was observed at later stage of SSF by strain DX-THS3 (Fig. 3C) instead of continuous decrease, and it maybe caused by the presence of high GUS activity which catalyzes GL to produce reducing sugar (glucuronic acid) at later stage of SSF (shown in Fig. 3B). Furthermore, the trend of total sugar also correlates with lignocellulose-degrading enzymes in SSF, high lignocellulose-degrading enzymatic activities reveal more utilization of carbon sources by microorganism, that means fast increase for the trend of total sugar.

**Table 1:** Analysis of lignocellulose-degrading enzymatic activities and production of GAMG under optimal fermentation conditions

	Enzymes	Max enzymatic activity (U/g)	Days (d)	*P*^a^ (U/g/d)	*P*^b^ (mg/g/d)
Non-optimization	CMCase	29.17 ± 1.01	10	2.93 ± 0.68	0.9 ± 0.03
	FPase	234.63 ± 4.28	7	33.52 ± 0.58	
	β-Glucosidase	6.81 ± 0.30	22	0.31 ± 0.02	
	Xylanase	72.52 ± 4.00	7	10.36 ± 0.44	
	GUSase	264.17 ± 12.17	20	13.21 ± 0.69	
Optimization	CMCase	33.67 ± 2.48	5	6.74 ± 0.88*	
	FPase	245.80 ± 13.40	3	81.93 ± 2.36*	
	β-Glucosidase	5.78 ± 0.69	20	0.29 ± 0.03	2.1 ± 0.07*
	Xylanase	83.44 ± 3.76	3	27.81 ± 1.78*	
	GUSase	271.42 ± 6.54	10	27.15 ± 2.06*	

^a^The production of lignocellulolytic enzymes when reaching maxim enzymatic activity

^b^The production of GAMG when the percent conversion of glycyrrhizin reaches 90%.

*P values analyzed by *t* test are statistically significant for a confidence level of 95% (*P* < 0.05)

From our results and older studies, one may conclude that excellent lignocellulose-degrading activities for utilization of complex substrates (usually crop straw) by microorganisms during SSF are needed (Ajijolakewu et al. 2016; Nutongkaew et al. 2019; Ezeilo et al. 2019). Thus, fungi that harbor rich genes coding lignocellulosic enzymes and excellent lignocellulose-degrading activities are usually used in SSF for the production of certain compounds in most previous reports; such fungi include *Ceratocystis paradoxa* TT1 (Nutongkaew et al. 2019) and *T. koningiopsis* TM3 (Nutongkaew et al. 2019) for the degradation of oil palm trunk to produce reducing sugar. *T. asperellum* UC1 (Ezeilo et al. 2019) utilizes raw oil palm frond leaves to produce cellulase and xylanase. Similarly, high lignocellulose-degrading enzymatic activities were found during SSF (Table 1 and Fig. 3D) using *C. globosum* DX-THS3 (Table 2). The 234.6 U/g FPase activity was detected during SSF, and this value is substantially higher than the other reported FPase activities in SSF using fungi. *T. asperellum* UC1 (Ezeilo et al., 2019) showed a relative high FPase (26.02 U/g) activity when using oil palm frond leaves as substrate for SSF, and most FPase activity was detected at 0.09–5 U/g (Table 2). High FPase activity indicates that complex cellulose can be degraded to generate easily hydrolyzed cello-oligomers, which are then further utilized by fungi. Furthermore, compared with the results of other studies, lignocellulose-degrading enzymes, including CMCase, xylanase, and β-glucosidase, from *C. globosum* DX-THS3 during SSF exhibited relative better enzymatic activities (Tables 1 and 2). Thus, high FPase activity, considerable CMCase, xylanase, and β-glucosidase activities were detected during SSF of *C. globosum* DX-THS3, which demonstrate wide application in SSF of strain DX-THS3. Our results also strongly consider *C. globosum* DX-THS3 as a potential producer for production of lignocellulose-degrading enzymes.

**Table 2:** Comparison of CMCase, FPase, β-glucosidase, xylanase, and GUS activities by *C. globosum* DX-THS3 and other fungi under SSF

Enzymes	Strains	Enzymatic activity (U/g)	Substrate	Reference
CMCase	*T. viridae* PAJ 01	64.56	Sugarcane bagasse/wheat bran	Natália et al. (2018)
	*Chaetomium* sp. TCF 01	12.13	Sugarcane bagasse/wheat bran	Natália et al. (2018)
	*A. fumigatus*	16.90	Wheat straw	Sherief et al. (2004)
	*Botryosphaeria* sp.	8.13	Empty fruit bunch	Bahrin et al. (2011)
	*Fomitopsis* sp. RCK2010	71.70	Wheat bran	Deswal et al. (2011)
	*Hypocrea nigricans* TT2	6.10	Oil palm trunk	Nutongkaew et al. (2019)
	*T. koningiopsis* TM3	7.13	Oil palm trunk	Nutongkaew et al. (2019)
	*T. asperellum* RCK2011	10.25	Wheat bran	Raghuwanshi et al. (2014)
	*T. asperellum* UC1	136.12	Oil palm frond leaves	Ezeilo et al. (2019)
	*C. globosum* DX-THS3	29.25	Licorice straw	This work
FPase	*Chaetomium* sp. TCF 01	0.09	Sugarcane bagasse/wheat bran	Natália et al. (2018)
	*A. fumigates*	0.98	Wheat straw	Sherief et al. (2004)
	*A. tubingensis* NKBP-55	3.80	Copra meal	Prajapati et al. (2018)
	*Botryosphaeria* sp.	3.30	Empty fruit bunch	Bahrin et al. (2011)
	*C. paradoxa TT1*	1.64	Oil palm trunk	Nutongkaew et al. (2019)
	*Fomitopsis* sp*. RCK2010*	3.50	Wheat bran	Deswal. et al. (2011)
	*T. aquaticus*	4.40	Wheat straw	Kalogeris et al. (2003)
	*T. asperellum* MR 1	0.72	Pressed oil palm petiole fiber	Ikubar et al (2018)
	*T. asperellumUC1*	26.02	Oil palm frond leaves	Ezeilo et al. (2019)
	*C. globosum* DX-THS3	234.60	Licorice straw	This work
β-Glucosidase	*Chaetomium* sp. TCF 01	3.81	Sugarcane bagasse/wheat bran	Natália et al. (2018)
	*A. tubingensis* NKBP-55	71	Copra meal	Prajapati et al. (2018)
	*I. obliquus*	2.58	Wheat bran	Xu et al. (2018a, b)
	*T. asperellum MR 1*	0.43	Pressed oil palm petiole fiber	Ikubar et al. (2018)
	*T. asperellum UC1*	130.09	Oil palm frond leaves	Ezeilo et al. (2019)
	*C. globosum* DX-THS3	6.42	Licorice straw	This work
Xylanase	*T. viridae* PAJ 01	351.74	Sugarcane bagasse/wheat bran	Natália et al. (2018)
	*Chaetomium* sp. TCF 01	39.75	Sugarcane bagasse/wheat bran	Natália et al. (2018)
	A.*niger* USM Al 1	35	Palm kernel cake	Kheng and Omar (2005)
	*A. fumigatus*	56.40	Wheat straw	Sherief et al. (2004)
	*A. tubingensis* TSIP9	59.30	Empty fruit bunch	Kitcha and Cheirsilp (2014)
	*A. tubingensis* NKBP-55	167	Copra meal	Prajapati et al. (2018)
	*T. koningiopsis* TM3	56.46	Oil palm trunk	Nutongkaew et al. (2019)
	*T. asperellum* MR 1	5.69	Pressed oil palm petiole fiber	Ikubar et al. (2018)
	*T. asperellum* UC1	255.01	Oil palm frond leaves	Ezeilo et al. (2018)
	*C. globosum* DX-THS3	72.52	Licorice straw	This work
GUSase	*A. terreus* Li-20	1.86a	–	Xu et al. (2018a, b)
	*Streptococcus* LJ-22	0.77a	–	Park et al. (2005)
	*P. purpurogenum.* Li-3	5.90 × 10^4^a	–	Zou et al. (2013)
	*C. globosum* DX-THS3	264.17b	Licorice straw	This work

^a^GUS proteins were purified and enriched, and enzymatic activities were detected

^b^GUS activity of solid-state medium was detected

### Popular carbon sources and nitrogen sources can significantly improve GAMG production by *C. globosum* DX-THS3

Nitrogen and carbon sources play key roles for production of specific products by fermentation using microorganism. Additional nitrogen and carbon sources were added to the licorice straw to further increase the productivity of GAMG by using SSF. First, the nitrogen source was optimized for GAMG production by SSF using *C. globosum* DX-THS3. NH_4_NO_3_, peptone, yeast powder, and yeast extract were used as nitrogen sources for the production of GAMG by *C. globosum* DX-THS3 (Additional file 1: Fig. S2A). Our results also show that the yield of GAMG was 1.44- (20.21 mg/g) and 1.19-fold (16.79 mg/g) higher than those of the control (14.02 mg/g) when using NH_4_NO_3_ and yeast extract as nitrogen sources after 20 days of fermentation, respectively. The GAMG yields were lower than that of the control when using peptone and yeast powder as nitrogen sources after 20 days of fermentation, with values reaching 13.17 and 13.37 mg/g, respectively. We further detected the GUS activity after adding NH_4_NO_3_, peptone, yeast powder, and yeast extract as nitrogen sources to the medium. Our results also showed the higher GUS activity when using NH_4_NO_3_ and yeast extract as nitrogen source than the control, whereas those obtained with peptone and yeast powder were lower after 20 days of fermentation (Additional file 1:Fig. S2B). Thus, NH_4_NO_3_ as additional nitrogen source can increase the yield of GAMG by *C. globosum* DX-THS3 in SSF. Meanwhile, the carbon source for GAMG production was investigated (Additional file 1: Fig. S3). The addition of fructose and glucose can produce 18.38 and 17.12 mg/g of GAMG after 20 days of fermentation (Additional file 1: Fig. S3A and 3B), respectively, which denoted increases of 33.9% and 24.7% than those obtained without a carbon source (13.73 mg/g). The addition of sucrose can generate 13.98 mg/g GAMG, but showed no significant effect on the production of GAMG using *C. globosum* DX-THS3 (Additional file 1: Fig. S3C). Among the carbon sources tested, lactose shows significant inhibitory effect, only 9.23 mg/g of GAMG was produced, which was a 34% reduction in GAMG compared with the control (Additional file 1: Fig. S3D). Furthermore, GUS activities were further detected after 20 days of SSF. Our results showed similar trend to those of GAMG production (Additional file 1: Fig. S3E). GUS activity with the addition of fructose was 338 U/g, which was significantly higher than that without carbon source (167 U/g). GUS activity with the addition of glucose was 1.5-fold (250 U/g, Additional file 1: Fig. S3F) higher than that without a carbon source (control, 167 U/g). Compared with the control, the addition of sucrose showed no significant effect on GUS activity (155 U/g, Additional file 1:Fig. S3G), but the addition of lactose significantly inhibited GUS activity (132 U/g, Additional file 1: Fig. S3H). Thus, NH_4_NO_3_ and fructose can significantly promote GAMG production of *C. globosum* DX-THS3 by SSF using licorice straw as a medium.

**Fig. 4:**
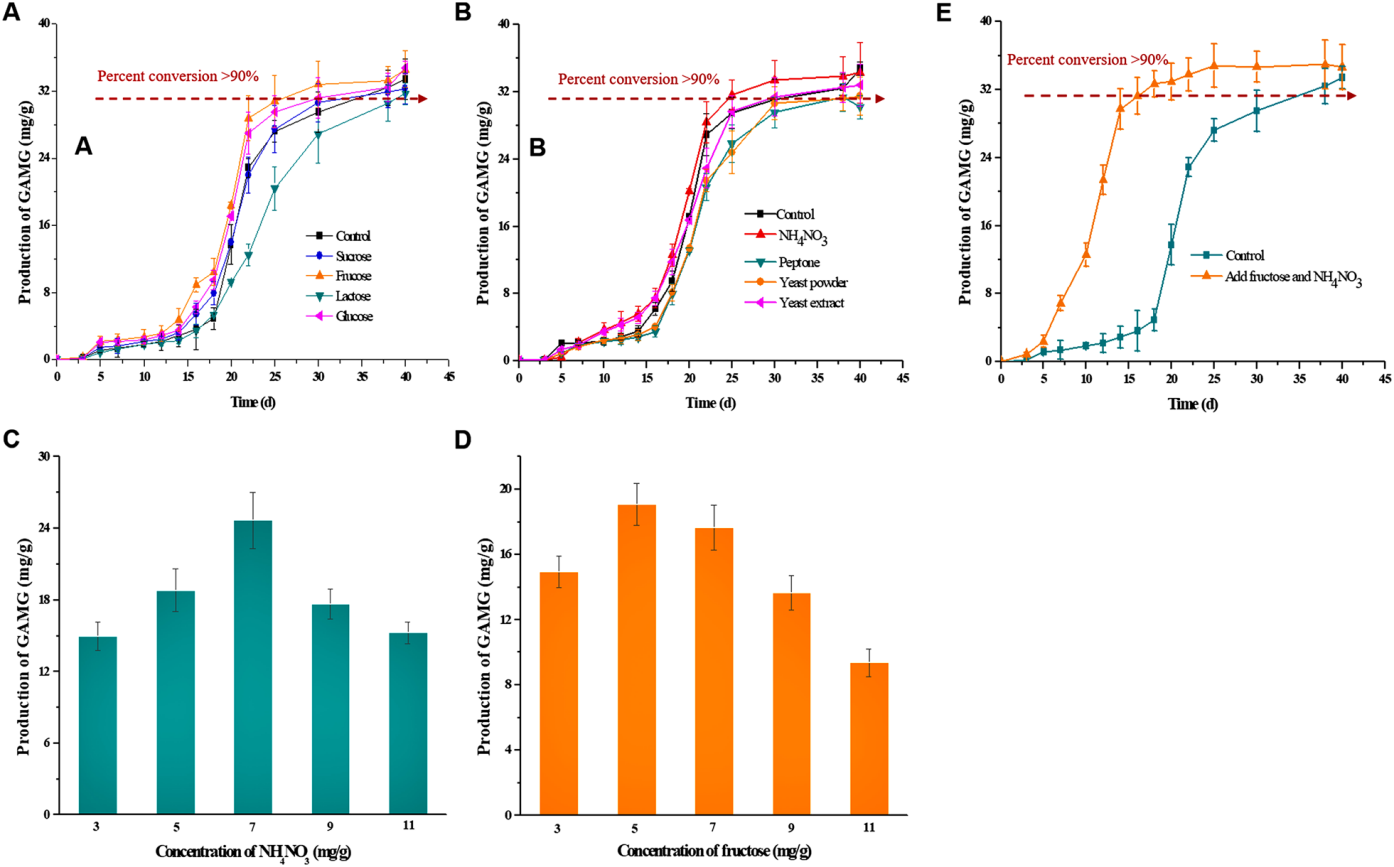
Effect of nitrogen and carbon sources on GAMG production. Yield of GAMG profile with the addition of **A** different nitrogen sources, **B** various carbon sources, **C** different concentration of NH_4_NO_3_, **D** different concentration of fructose and **E** under optimal fermentation conditions

The yield of GAMG during the SSF period with or without carbon and nitrogen sources was detected to further investigate the production of GAMG using *C. globosum* DX-THS3. First, we detected the yield of GAMG with or without extra carbon source, respectively (Fig. 4A). Similar variation trends of GAMG yields were observed. All the test yields of GAMG slowly increased at the initial stage of SSF (0–15 days), whereas those at the middle stage rapidly increased (15–30 days). Then, the yields of GAMG stabilized at about 32 mg/g at the late stage of SSF (after 30 days). Although all the test yields of GAMG exhibited no significant difference at the later stage of SSF (Additional file 1: Fig. S4), the productivities of GAMG by SSF with different carbon sources or without a carbon source presented significant differences. Compared with the control (33 days), the addition of fructose was the fastest for GAMG production, and 25 days of SSF was used to reach 90% conversion (*Y* = 31.52 mg/g), 28 days for the addition of glucose, and 30 days for the addition of sucrose. The addition of lactose to the medium can inhibit GAMG production by *C. globosum* DX-THS3 in SSF, requiring 38 days to reach 90% conversion. Meanwhile, the addition of nitrogen source also showed significant effect on the production of GAMG by SSF using *C. globosum* DX-THS3. As shown in Fig. 4B, compared with that without nitrogen source, the addition of NH_4_NO_3_ to the medium achieved improved production of GAMG by *C. globosum* DX-THS3, with a reduction of 9 days in the production period to transform 90% of GL compared with that without a nitrogen source. Yeast extract showed no effect on the production of GAMG, whereas yeast powder and peptone can inhibit GAMG production. Thus, NH_4_NO_3_ and fructose were further optimized. First, different concentrations of NH_4_NO_3_ and fructose (3, 5, 7, 9, and 11 mg/g) were added to the SSF medium. Then, GAMG production was detected after 20 days of SSF. Our results show that 7 mg/g NH_4_NO_3_ and 5 mg/g fructose were the optimum conditions for GAMG production by *C. globosum* DX-THS3 using SSF (Fig. 4C and D, respectively). Based on the above results, GAMG was produced faster under these optimal conditions than the control: 0.26–0.85 mm particle size, 28 °C, 96 h seed age, 20% inoculum size, 1:3 solid–liquid ratio, 7 mg/g NH_4_NO_3_, and 5 mg/g fructose (Fig. 4E). Under these optimal conditions, the percent conversion of GL reached 90% within 15 days, whereas the control needed 35 days, that is, an additional 20 days needed for 90% conversion (Table 1 and Fig. 4E). The productivity of optimization (*P* = 2.1 mg/g/day) increased by 133.33% compared with that in non-optimized conditions (*P* = 0.9 mg/g/day). Furthermore, the productivities of CMCase, FPase, xylanase and GUS activity were better than the control (Table 1).

**Fig. 5:**
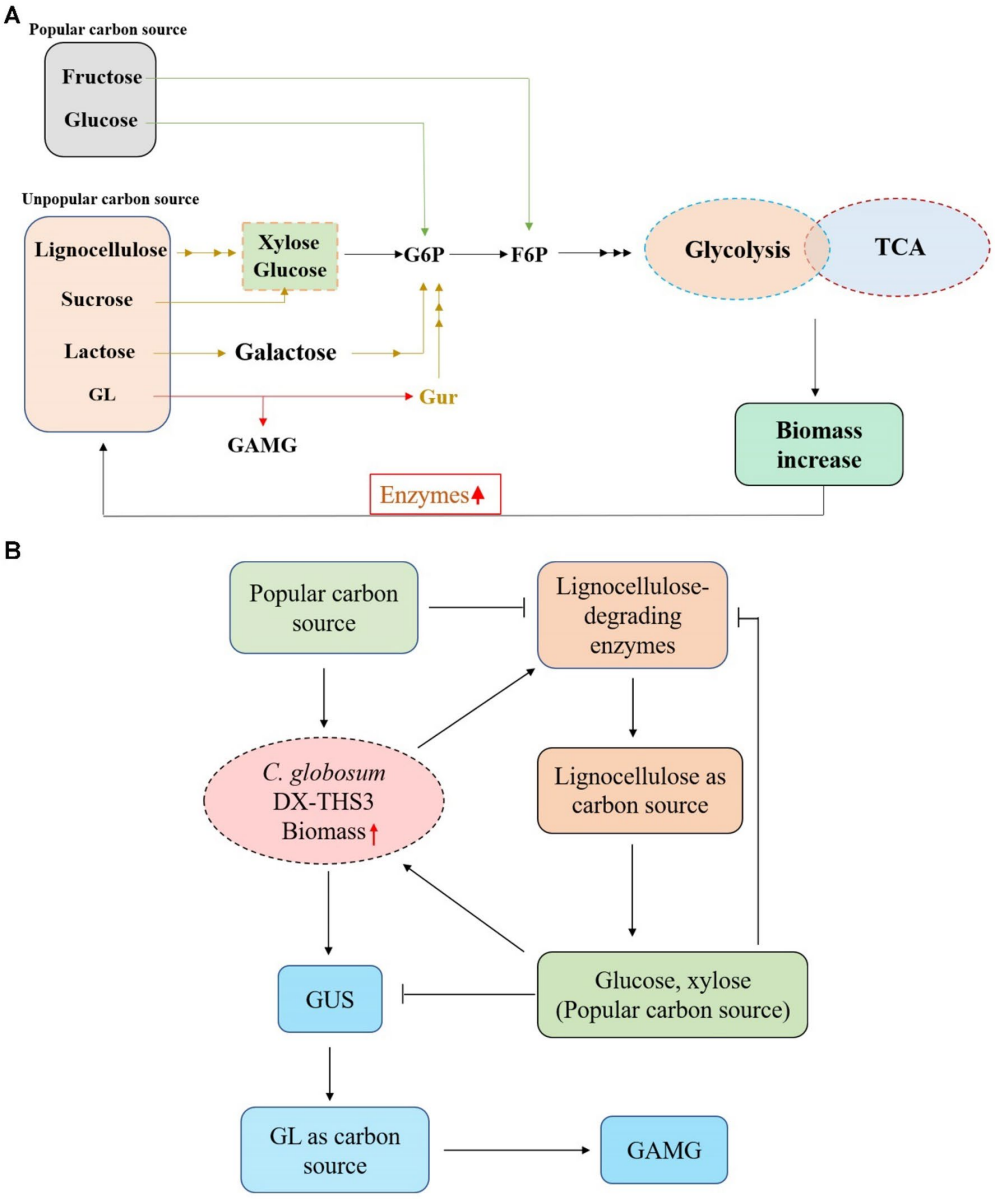
Predicted mechanism for promoting GAMG production by the addition of fructose to the medium. **A** Fructose and glucose (popular carbon sources) were more rapidly utilized by *C. globosum* DX-THS3 than lignocellulose, sucrose, lactose, and GL because such popular carbon sources can directly enter glycolysis and TCA cycle. **B** Popular carbon sources can promote the rapid accumulation of *C. globosum* DX-THS3 biomass, producing more lignocellulose-degrading enzymes, rapidly utilizing lignocellulose, and finally fast-transforming GL into GAMG

Microorganic fermentation can be classified into three stages: early, middle, and late stages. In general, low-cost and easily available substrates, such as crop straw, are usually used as medium for SSF using fungi. However, complex and adequate enzymes are needed for the degradation of polysaccharides in these substrates (Prajapati et al. 2018; Deswal et al. 2011; Pandey 2003; Nutongkaew et al. 2019; Ezeilo et al. 2019). Compared with liquid fermentation, more time is needed for fungal cell growth to secrete a complex enzyme system in SSF. Thus, fast accumulation of biomass is the key for shortening the early stage period and increasing the productivity of SSF. To reduce this period, we first optimized the fermentation conditions, including the particle size, temperature, seed age, inoculum size, and water content. However, the early stage of GAMG production was long (about 25 days) (Fig. 3B). Thus, several carbon sources that are easily utilized by microorganisms were considered. Glucose, fructose, and several monosaccharides (popular carbon sources) can be directly entered into glycolysis and TCA cycle for fungal growth (Fig. 5A). Therefore, we considered adding these carbon sources to the medium to increase productivity when using microorganisms for SSF. Our results demonstrate that the early stage period of SSF was reduced by about 10 days when adding fructose and NH_4_NO_3_ to the medium, thus significantly increasing productivity (Table 1 and Fig. 4E). In this study, lignocellulose and GL of licorice straw were used as carbon sources for *C. globosum* DX-THS3 growth, and high lignocellulose-degrading enzymatic activities were detected during 5–15 days of fermentation (Fig. 3D). High GUS activities were observed at 20–25 days (Fig. 3C), and the concentration of reducing sugar abruptly increased at 20 days of fermentation. These findings demonstrate that lignocellulose was first utilized, followed by GL, by *C. globosum* DX-THS3. Thus, certain carbon sources were added to the medium to reduce the early stage period and increase the GAMG production. In summary, at the initial period of SSF, several lignocellulose-degrading enzymes were secreted because of the low biomass of *C. globosum* DX-THS3. *C. globosum* DX-THS3 slowly grew. Thus, a long period was needed for the accumulation of *C. globosum* DX-THS3 to secrete sufficient lignocellulose-degrading enzymes. Subsequently, GUS was rapidly secreted by *C. globosum* DX-THS3 for the utilization of GL as a carbon source to generate GAMG and glucuronic acid (Gur). If several popular carbon sources, such as fructose, are added to medium, *C. globosum* DX-THS3 will grow fast, which can promote the secretion of lignocellulose-degrading enzymes and fast utilization of lignocellulose, significantly reducing the time for production of GAMG (Fig. 5B).

Although there are important discoveries revealed by this work, there are also limitations. First, we cannot determine fructose and NH_4_NO_3_ whether is the optimal carbon and nitrogen source, and more studies need to be further investigated. Second, the method for separation of GAMG should be investigated. Furthermore, one important future direction of GAMG production is successional production of GAMG using fed-batch fermentation. Notwithstanding its limitation, this study does suggest a novel, fast, and low-cost method for the production of concurrent GAMG and lignocellulolytic enzymes.

## Conclusions

Compared to chemical methods, the biotransformation of GL into GAMG catalyzed by GUS is an environmentally friendly approach due to the mild reaction conditions and the high yield of GAMG (Michaelis et al. 2011; Ito et al. 1988; Huang et al. 2016; Wang et al. 2015a). GUS was mainly obtained by fungal fermentation and it is very tedious and costly. Thus, efficient approaches for large-scale GAMG production need to be developed. In our previous studies, an endophytic fungus *C. globosum* DX-THS3 was isolated from Dongxiang wild rice which harbors a GUS with specificity and highly transformable GL to generate GAMG and rich genes coding lignocellulose-degrading enzymes (Wang et al. 2015b; Zhang et al. 2020). Based on our previous results, we aimed to investigate the feasibility of GAMG production by *C. globosum* DX-THS3 via SSF and provide an efficient strategy for large-scale production of GAMG in this study. First, *C. globosum* DX-THS3 was cultured on licorice straw for SSF. After 20 days of SSF, GAMG was obtained and GUS activity was detected. Then, optimization of fermentation conditions was performed; however the fermentation period for GAMG production was too long (transformation of 90% GL to GAMG need about 35 days). To reduce the fermentation period, extra carbon and nitrogen sources were added into medium for SSF. Our results showed that the fermentation period was significantly reduced when fructose was added into the medium. Under the optimal fermentation conditions, 90% GL was transformed to GAMG within 15 days, whereas the control needed 35 days. The productivity of GAMG can reach 2.1 mg/g/day, which was 2.33-fold higher than that of the control. Meanwhile, high lignocellulose-degrading enzymatic activities were also detected during SSF. Therefore, our study contributes to the application of SSF in the production of GAMG and strongly demonstrated *C. globosum* DX-THS3 as a potential candidate for producing lignocellulose-degrading enzymes.

### Supplementary Information

**Additional file 1: Fig. S1.** The schematic map of hydrolyzing GL into GAMG and GA. **Fig. S2.** Optimization of nitrogen source, including NH_4_NO_3_, peptone, yeast powder, and yeast extract. NH_4_NO_3_ and yeast extract can significantly increase the production of GAMG (A) and GUS activity (B) during SSF by *C. globosum* DX-THS3. **: p < 0.01, *: p < 0.05. **Fig. S3.** Effect of carbon sources, including fructose, glucose, sucrose, and lactose, on the production of GAMG (A–D) and GUS activity (E–H) after 20 days of SSF using *C. globosum* DX-THS3. **: p < 0.01, *: p < 0.05. **Fig. S4.** Effect of nitrogen and carbon sources on GAMG production.

## Data Availability

Not applicable.
