# Supplementary material for: Concurrent production of glycyrrhetic acid 3-O-mono-β-d-glucuronide and lignocellulolytic enzymes by solid-state fermentation of a plant endophytic Chaetomium globosum
Source: Bioresour Bioprocess. 2021 Sep 15;8(1):88. doi: 10.1186/s40643-021-00441-y (PMC8442819; doi:10.1186/s40643-021-00441-y)
Supplement: Supplementary file 1 — Additional file 1: Fig. S1. The schematic map of hydrolyzing GL into GAMG and GA. Fig. S2. Optimization of nitrogen source, including NH4NO3, peptone, yeast powder, and yeast extract. NH4NO3 and yeast extract can significantly increase the production of GAMG (A) and GUS activity (B) during SSF by C. globosum DX-THS3. **: p < 0.01, *: p < 0.05. Fig. S3. Effect of carbon sources, including fructose, glucose, sucrose, and lactose, on the production of GAMG (A–D) and GUS activity (E–H) after 20 days of SSF using C. globosum DX-THS3. **: p < 0.01, *: p < 0.05. Fig. S4. Effect of nitrogen and carbon sources on GAMG production. [file 40643_2021_441_MOESM1_ESM.docx]

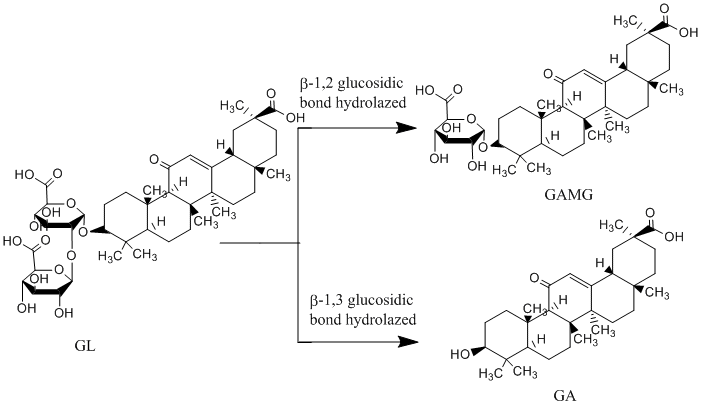


**Fig. S1** The schematic map of hydrolyzing GL into GAMG and GA.


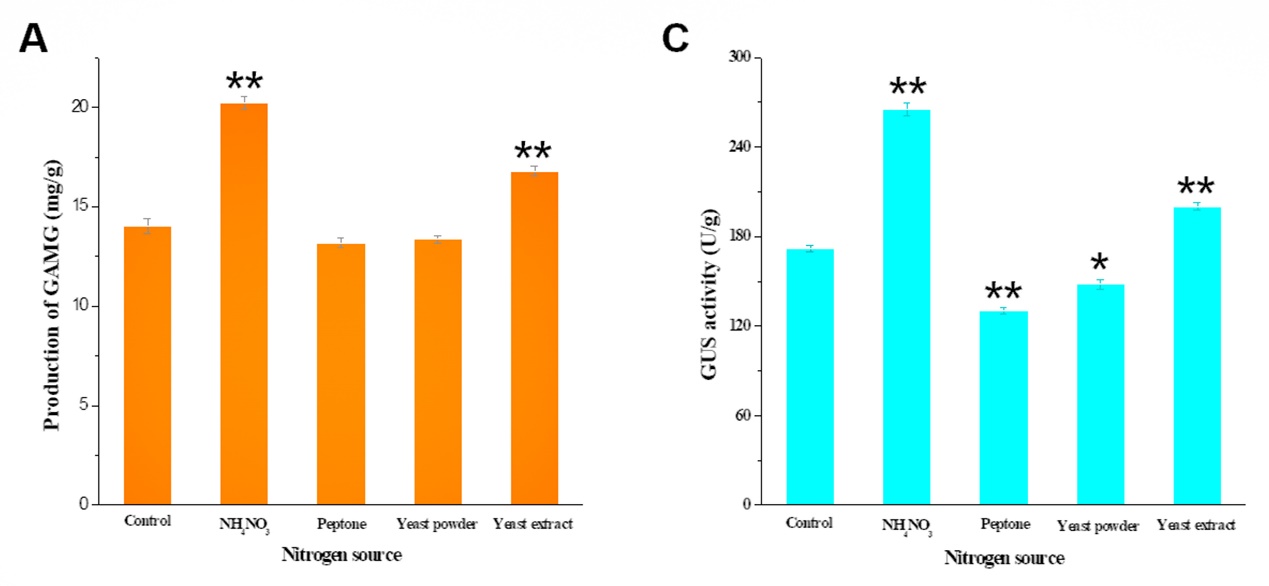


**Fig. S2** Optimization of nitrogen source, including NH_4_NO_3_, peptone, yeast powder, and yeast extract. NH_4_NO_3_ and yeast extract can significantly increase the production of GAMG (A) and GUS activity (B) during SSF by *C. globosum* DX-THS3. ******: p<0.01, *****: p<0.05.


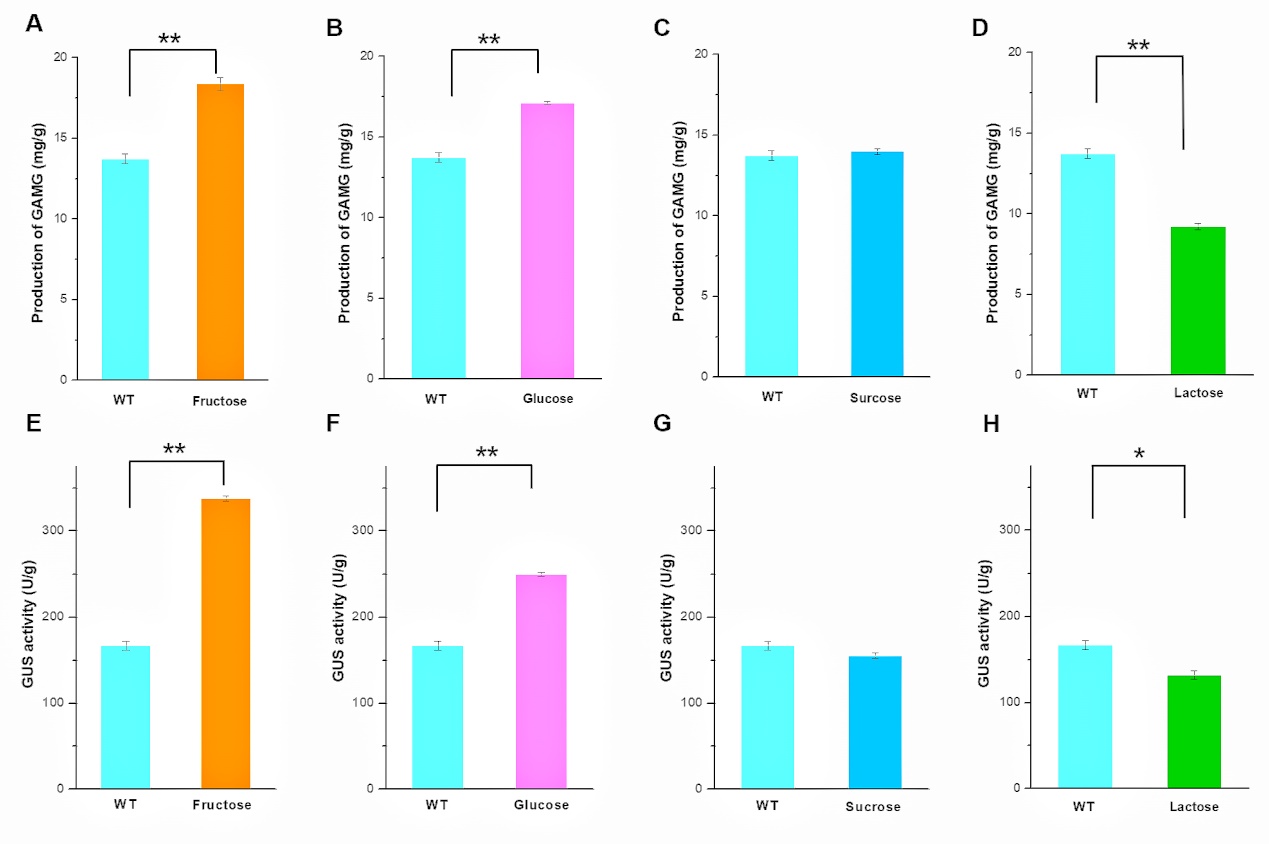


**Fig. S3** Effect of carbon sources, including fructose, glucose, sucrose, and lactose, on the production of GAMG (A–D) and GUS activity (E–H) after 20 days of SSF using *C. globosum* DX-THS3. ******: p<0.01, *****: p<0.05.


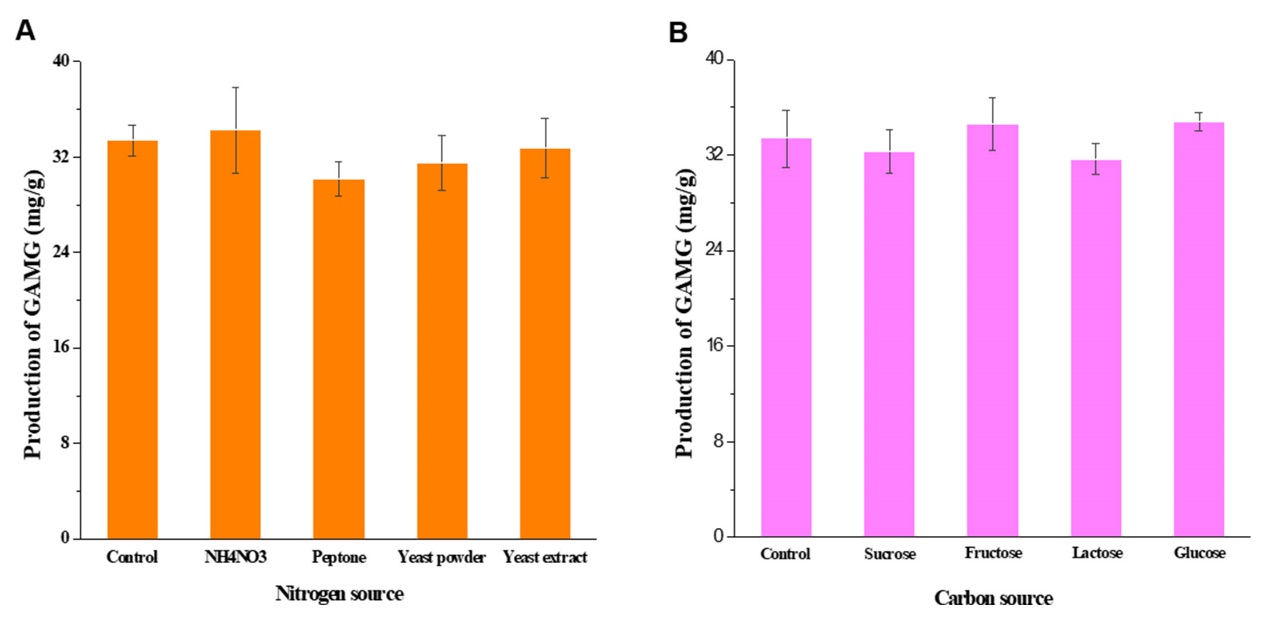


**Fig. S4** Effect of nitrogen and carbon sources on GAMG production.
